# Infertility treatment outcome in sub groups of obese population

**DOI:** 10.1186/1477-7827-7-52

**Published:** 2009-05-27

**Authors:** Khalid A Awartani, Samar Nahas, Saad H Al Hassan, Mashael A Al Deery, Serdar Coskun

**Affiliations:** 1 Reproductive Medicine, Department of Obstetrics & Gynecology, King Faisal Specialist Hospital and Research Centre, Riyadh, Saudi Arabia; 2 Alfaisal Collage of Medicine, Alfaisal University, Riyadh, Saudi Arabia; 3 Department of Pathology and Laboratory Medicine, King Faisal Specialist Hospital and Research Centre, Riyadh, Saudi Arabia

## Abstract

**Background:**

Obesity is a common disorder with a negative impact on IVF treatment outcome. It is not clear whether morbidly obese women (BMI >= 35 kg/m2) respond to treatment differently as compared to obese women (BMI = 30–34.9 kg/m2) in IVF. Our aim was to compare the outcome of IVF or ICSI treatments in obese patients to that in morbidly obese patients.

**Methods:**

This retrospective cohort study was conducted in a tertiary care centre. Patients inclusion criteria were as follows; BMI ≥ 30, age 20–40 years old, first cycle IVF/ICSI treatment with primary infertility and long follicular pituitary down regulation protocol.

**Results:**

A total of 406 obese patients (group A) and 141 morbidly obese patients (group B) satisfied the inclusion criteria. Average BMI was 32.1 ± 1.38 kg/m2 for group A versus 37.7 ± 2.99 kg/m^2 ^for group B. Patient age, cause of infertility, duration of stimulation, fertilization rate, and number of transferred embryos were similar in both groups. Compared to group A, group B had fewer medium size and mature follicles (14 vs. 16), fewer oocytes collected (7 vs. 9) and required higher doses of HMG (46.2 vs. 38.5 amps). There was also a higher cancellation rate in group B (28.3% vs. 19%) and lower clinical pregnancy rate per started cycle (19.9% vs. 28.6%).

**Conclusion:**

In a homogenous infertile and obese patient population stratified according to their BMI, morbid obesity is associated with unfavorable IVF/ICSI cycle outcome as evidenced by lower pregnancy rates. It is recommended that morbidly obese patients undergo appropriate counseling before the initiation of this expensive and invasive therapy.

## Background

Obesity is considered to be the greatest nutritional problem in the industrialized world [1,2]. The World Health Organization (WHO) and the National Institutes of Health (NIH) define obesity as the body mass index (BMI) of ≥ 30 kg/m^2^. Obesity is further divided into three categories; class I obesity with BMI = 30–34.9 kg/m^2^, class II with BMI = 35–39.9 kg/m^2^, and class III with BMI ≥ 40 kg/m^2 ^[1,2]. This classification corresponds to increasing health risks of the obesity related comorbidities.

In Saudi females at childbearing age, obesity is among the most frequently encountered multifactorial disorders and it has been reported that 38% of females attending a referral in vitro fertilization (IVF) program suffers from obesity [3,4].

Multiple reproductive dysfunctions have been associated with obesity including anovulation, and infertility [5]. Obese patients undergoing IVF or intracytoplasmic sperm injection (ICSI) treatment are known to have increased FSH requirement, fewer collected oocytes, and frequent cycle cancellation, lower pregnancy rate and increase miscarriage rate than their non-obese counterpart [6-15].

In the literature, it is unclear whether response and outcome of women who are morbidly obese (class II and III obesity; BMI ≥ 35 kg/m^2^) differ from their class I (BMI 30–34.9) counterparts in IVF or ICSI treatment cycle.

The objective of this study is to compare the outcome of the IVF or ICSI treatment in obese patients to that in the morbidly obese population.

## Discussion

### Methods

Patients who were 20–40 years old, had no living children, and underwent their first cycle of IVF/ICSI treatment with long follicular pituitary down regulation protocol followed by controlled ovarian stimulation at King Faisal Specialist Hospital and Research Centre, Riyadh, Saudi Arabia between January 2000 to December 2003 were included in this retrospective cohort study.

Ovarian stimulation, oocyte retrieval, semen preparation, embryo culture, and embryo transfer protocols have been performed as previously published [16]. Briefly, pituitary suppression was achieved by gonadotrophin-releasing hormone agonist long protocol by IM. injection of 1.875 mg Lupron Depot (Abbott Laboratories, Chicago, Illinois, USA) on day 3 of the menstrual cycle. Twenty-one days after the Lupron injection, ovarian stimulation was initiated with 225 IU (3 ampoules) daily IM. injection of human menopausal gonadotrophin (Menegon; Ferring, Germany) and adjusted according to patients' response. Ten thousand IU of human chorionic gonadotrophin (HCG) (Pregnyl, Organon, Oss, the Netherlands) were given IM. When three or more follicles reached 18 mm in diameter, oocyte retrieval was performed with transvaginal ultrasound guidance under IV sedation 36 h after HCG injection. The follicular aspirate was poured into 60 mm Falcon dishes (Beckton Dickinson Labware, Franklin Lakes, New Jersey, USA) and cumulus-oocyte complexes were transferred into another dish with Medi-Cult flush medium (Medi-Cult, Jillinge, Denmark). Each complex was evaluated for maturity based on cumulus-corona cell morphology. Cumulus-oocyte complexes were transferred into IVF medium (Medi-Cult). Fertilization was achieved by IVF or ICSI. On day 3, the two best by morphology dividing embryos (if available) were transferred under ultrasound guidance. Patients were supplemented with progesterone (100 mg/daily IM, Steris Laboratories Inc., Phoenix, Arizona, USA). Pregnancy was first confirmed with a Tandom Icon urine HCG test (Hybritech, San Diego, California, USA) and serum βHCG concentrations at 13 days, and by ultrasound 5 weeks after embryo transfer. The pregnancies reported in this study are clinical pregnancies where fetal heart beat was positive by transvaginal ultrasound scan at 7^th ^gestational week, so all biochemical pregnancies (when a previously positive pregnancy test became negative before ultrasonographic detection of an embryonic sac in the fifth week of pregnancy or later), and preclinical losses (if the loss occurred after the gestational sac is seen but before the fetal heart activity is seen) were excluded.

### Statistics

Patients were divided into two groups: group (A) included obese women with BMI 30–34.9 kg/m^2^; group (B) included morbidly obese women with BMI ≥ 35 kg/m^2^. Clinical pregnancy per started cycle was used as the primary outcome. Secondary outcomes included duration of stimulation, cycle cancellation, number of oocyte retrieved, and fertilization rate.

Statistical analysis was performed using S-plus 2000. A two-tailed t-test was used for parametric data, a Mann-Whitney test for non-parametric data, and Chi-square test for binomial data. P < 0.05 was considered statistically significant.

## Results

During the study period, 1545 women aged 20–40 years old had their first stimulation cycle using long follicular pituitary down regulation protocol. In this population, 547 women (35%) had a BMI ≥ 30 kg/m^2^. Of those 406 women were obese, and 141 women were morbidly obese. Average BMI in the obese patients was 32.1 ± 1.38 kg/m^2^, and 37.7 ± 2.99 kg/m^2 ^for the morbidly obese ones (p < 0.0001). Obese and morbidly obese women had similar age, 30.6 ± 4.57 vs. 31.6 ± 5.1 years, respectively. Both groups had similar primary causes of infertility (Table 1).

**Table 1:** Patients' characteristics.

	Group A (n = 406)	Group B (n = 141)	P value
Age (years)	30.6 ± 4.57	31.6 ± 5.1	NS
BMI (kg/m^2^)	32.1 ± 1.38	37.7 ± 2.99	<0.0001
Male factor (%)	265 (65.3%)	84 (59.6%)	NS
Unexplained infertility (%)	45 (11.1%)	19 (13.5%)	NS
Tubal Factor (%)	86 (21.2%)	32 (22.7%)	NS
Ovulatory cycle	211 (52%)	63 (45%)	NS
ICSI (%)	330 (81.3%)	118 (83.7%)	NS

Note: Data are presented as mean ± SD unless other wise indicated, NS: P value is > 0.05.

Table 2 shows the comparison of treatment outcome between the two groups. Duration of stimulation was similar in both groups, but the morbidly obese women required much higher doses of HMG for stimulation. Morbidly obese women also had a lower number of medium size (between 11–17 mm) and mature follicles (≥ 18 mm). In addition there was a higher cancellation rate per started cycle for the morbidly obese women (28.3% vs. 19%; P = 0.02). The cycle cancellation was either due to poor ovarian response to gonadotropin stimulation (less than 3 mature follicles) or increased risk of ovarian hyperstimulation syndrome. The rate of sever hyperstimulation syndrome was less than 1% in both groups.

**Table 2:** Comparison of cycle's outcome between obese and morbid obese patients.

Cycle outcome	Group A (n = 406)	Group B (n = 141)	P value
Number of follicles	16 (9–22)	14 (7.5–19.5)	0.046
Number of oocyte collected	9 (4–14)	7 (3–13)	0.027
Number of fertilized oocytes	5 (2–8)	4 (1–7)	NS
*Number of embryos transferred:*			
*1*	*30 (9%)*	*14 (14%)*	*NS*
*2*	*299 (91%)*	*88 (86%)*	*NS*
Length of stimulation	14 (12–15)	14 (12–16)	NS
Dose of HMG	36 (30–45)	44 (36–56)	0.047
Cancellation rate n (%)	77(19%)	40(28.3%)	0.02
Clinical Pregnancy rate n (%)	116(28.6%)	28(19.9%)	0.042
Preclinical loss	18 (4.4%)	8 (5.7%)	NS
Biochemical pregnancy	8 (2%)	4 (2.8%)	NS

Note: Data are presented as median (lower and upper quartile) unless other wise indicated.

In this study, the number of patients who reached embryo transfer or had more than one embryo to be selected for transfer were higher in obese patients compared to those who were morbidly obese (74% vs 62% with P = 0.025). When compared to obese patients, morbidly obese women also had lower clinical pregnancy rate per started cycle (19.9% vs. 28.6%; p = 0.042).

The negative impact of obesity in the outcome of assisted reproductive technology has been suggested by multiple reports [6-15]. In these previous reports, the comparison was made to the outcome achieved in women with normal body mass index. In this study, we report the outcome of ART treatment for morbidly obese patients and compared the outcome to their obese counterpart. The study population was relatively homogenous, with comparable age and cause of infertility. The treatment protocol was standardized and all women were undergoing their first treatment cycle. Our results suggest that morbidly obese women have a lower pregnancy rate per started cycle when compared to obese patients. This could be explained by the fact that these women would have lower number of medium size and mature follicles, and also had lower number of oocyte retrieved, giving less choice in the selection of best embryos for transfer based on morphological criteria.

While this study was not designed to study the mechanism of the less favorable outcome in morbidly obese population, data from Butzow et al. [17] and Gurbuz et al [18] showing a positive correlation between BMI and the leptin serum level during ovarian stimulation in IVF cycles, and a negative correlation between increased levels of leptin and the number of oocyte retrieved, suggests that the main source of leptin is the adipose tissue. It is possible that high leptin concentration acting at the ovarian level suppresses the ovarian response to gonadotropins. These findings are consistent with our findings since the morbidly obese women had a lower number of oocyte retrieved. The insulin resistance, hyperinsulinemia, and hyper androgeniemia increased with increasing BMI [5]. These hormonal changes might affect the ovarian response, oocyte quality, embryo quality and/or endometrial receptivity as previously suggested [13,14,19,20]. In this study, the lack of ovarian response and availability of good quality embryos for transfer was the predisposing factor for the lower pregnancy rate seen in morbidly obese population.

A limitation of this study is that the live birth rate could not be assessed due to our inability to complete follow up with these patients after 7 weeks of gestation; most of these patients receive care for their pregnancy in remote areas. Additionally it would be interesting to know the live birth rate per started cycle and the late miscarriage rate (following +ve fetal heart beat) for the morbidly obese population in comparison to the obese patients.

This is the largest published dataset describing the outcome of morbidly obese women undergoing IVF/ICSI treatment using the obese population as the control. One of the important findings of this study was that the probability of cancellation per started cycle in morbidly obese patients due to risk of ovarian hyperstimulation syndrome (OHSS), or due to lack of ovarian response was higher than the probability of clinical pregnancy.

As other authors have suggested, weight loss might improve the reproductive function of the obese population [5,21-24]. We suggest that life style modification and weight loss programs should be advised for the morbidly obese women before they attempt IVF/ICSI treatment. This in concordance with new recommendations of the British Fertility Society recommendations to defer treatment in obese women until a BMI of <35 is reached [25].

## Summary

In a homogeneous infertile and obese population stratified according to their BMI, morbidly obese women undergoing IVF or ICSI therapy have lower clinical pregnancy rate when compare to obese patients. We would suggest that before initiation of this expensive and invasive therapy in morbidly obese population, couples should receive counseling about the expected performance and the anticipated clinical pregnancy rate per started cycle.

## Competing interests

The authors declare that they have no competing interests.

## Authors' contributions

KAA participated in the design of the study, data collection, statistical analysis, and manuscript drafting. SN participated in the design of the study and data collection. SAH participated in the design of the study and manuscript drafting. MAD participated in the design of the study and data collection. SC participated in the design of the study, data collection, statistical analysis, and manuscript drafting. All authors read and approved the final manuscript.
